# A Cross-Sectional Study Exploring the Role of Social Isolation in the Relationship Between Food Insecurity, Depressive Symptoms, and Resource Use Among Midwestern Rural Veterans in the U.S.

**DOI:** 10.3390/nu17020318

**Published:** 2025-01-16

**Authors:** Mwiza A. Uwashimimana, Shelley MacDermid Wadsworth, Douglas A. Sneddon, Jake Newton, Heather A. Eicher-Miller

**Affiliations:** 1 Department of Nutrition Science, Purdue University, West Lafayette, IN 47907, USA; muwashim@purdue.edu; 2 Department of Human Development and Family Science, Purdue University, West Lafayette, IN 47906, USA; shelley@purdue.edu (S.M.W.); dsneddon@purdue.edu (D.A.S.); newton24@purdue.edu (J.N.)

**Keywords:** food security, social isolation, depression

## Abstract

Background/Objectives: The study’s objective was to determine whether social isolation serves as a mediator in the cross-sectional relationship between food insecurity, both as a short-term and longer-term situation, with resource use and depressive symptoms as outcomes. Methods: This cross-sectional design study utilized secondary survey data, including 30-day and 12-month food security measured by the U.S. Household Food Security Survey Module. The Baron–Kenny mediation approach was used to determine whether social isolation mediated the relationship between food security, depressive symptoms, and resource use (*p* ≤ 0.05). Results: Social isolation mediates the association between both 30-day and 12-month food security with depressive symptoms but not resource use. Conclusions: Acknowledging and targeting social isolation, policies, and interventions that integrate peer support and community outreach to promote food security could support rural veterans food security and mental health.

## 1. Introduction

Food insecurity is a public health concern in the U.S. where there are not enough resources for food; food insecurity affects different groups, based on their characteristics or experiences, disproportionately [1]. One of these groups is U.S. veterans. There are over 16 million veterans in the U.S., a term defining those who have served in the active military, naval, or air service without a dishonorable release [2]. Most are white (74.2%), male (89.7%), over age 65 (49.3%), with a 30% disability rate compared to 16% in the general population [3]. Approximately 7.5% of veterans live in poverty compared with 11.6% in the general population [3] and on average, 11.1% of veterans are food insecure [4]. Veterans living in rural areas have a food insecurity rate of 13.9% while the rate in urban areas is 10.6% [5]. Food insecurity among rural veterans may be further exacerbated by a higher prevalence of disability and lower employment rate compared to their urban counterparts [5]. Previous literature has shown those experiencing food insecurity often have a higher share of poor chronic health outcomes, such as obesity and diabetes, compared with those who are food secure [6,7].

Food insecurity has also been consistently associated with poor mental health outcomes, including depression, an outcome that is also experienced by high rates of veterans. Food insecurity among veterans shows a 3 times more likely report of symptoms of depression compared with food security [7,8]. Rural veterans had a slightly higher rate of reporting depression (1.12 times) than urban veterans [9]. Residing in rural areas may come with healthcare challenges in terms of provider shortages, fewer healthcare facilities, and closure of community hospitals due to financial instability, diminishing the accessibility of mental healthcare for rural veterans [10]. Social isolation, a condition of physical isolation that limits the development and expansion of social networks, leading to limited contact with other people and the community [11], is also linked to depression among veterans [12] and may be a factor differentiating the experience of food insecurity and the link to poor mental health outcomes, including depression. For example, food insecurity is associated with social isolation; U.S. adults who experienced food insecurity reported greater social isolation compared to those who were food secure [13]. The stigma that comes with the inability to feed one’s family adequate food or the use of assistance programs such as food banks [14] and the unique barriers of residing in rural areas, specifically geographic isolation, limited transportation, and limited availability of social services [5], may be contributors to difficulties in establishing social networks that may lead to social isolation among this population. Social networks provide social support, which may be helpful in providing information about assistance programs and offering rides to food banks, mental health clinics, and other social services [15], buffering the stress of food insecurity and depression.

Food assistance programs where individuals and households receive a benefit that allows them to acquire additional foods on the basis of income, along with federal assistance programs that offer benefits based on income, and veterans’ disability programs that similarly provide benefits based on disability due to a service-related injury, are resources that may be utilized by veterans and help them to maintain food security. More specifically, the Supplemental Nutrition Assistance Program (SNAP) for household eligibility at 130% of the poverty income level or below is one such program [16] that provides a voucher to acquire food, while the Temporary Assistance for Needy Families (TANF) offers financial assistance to families with children based on income [17], and the Veteran Affairs (VA) disability compensation provides supplemental income to veterans with service-connected illness or injury recognized by the VA [18]. These programs and other programs that support veterans through benefits, supplemental income, or food, and the use of these programs, defined as “resource use” in this paper, may extend resources to veterans that could be particularly helpful to those in food-insecure situations; for example, these resources may provide additional access to food or other needs to free up other resources for food. However, veterans may feel reluctant to use such services since the military culture emphasizes strength and self-reliance, making it difficult for them to seek help [19,20].

**Figure 1:**
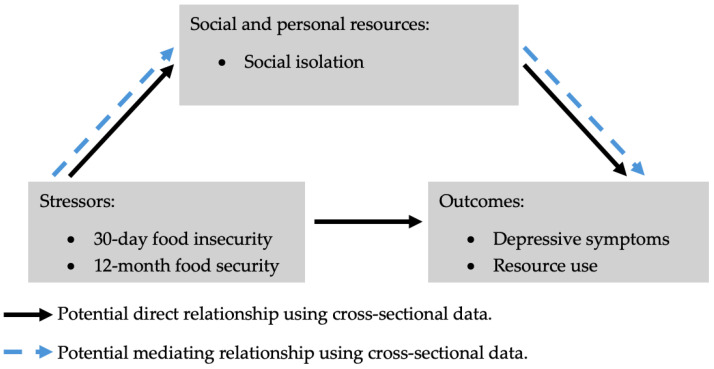
Depiction of the potentially mediating relationship of social isolation differentiating the link of food insecurity with depressive symptoms and resource use through the Stress Process Model-derived framework (Adapted from https://www.jstor.org/stable/2136676?origin=crossref, accessed on 1 December 2024).

The relationships of food insecurity, social isolation, depression, and resource use among veterans are poorly understood. The Stress Process Model (SPM) is a theoretical framework that may be helpful in explaining the relationships between stressors such as food insecurity, with social isolation as a potential mediator, and outcomes such as resource use, including assistance programs, and depressive symptoms as a mental health outcome [21]. Social isolation may act as a statistical mediator, whereby the relationship of food insecurity with depressive symptoms may be accounted for by social isolation status when evaluated on cross-sectional data. For instance, food-insecure veterans may be more likely to report depressive symptoms when they are socially isolated, and similarly, food-insecure veterans may be less likely to use resources when they are socially isolated. Furthermore, these relationships may differ based on the time frame of food insecurity or whether food insecurity was reported as experienced in the short term, over the past 30 days, or over a longer period of time, during the past 12 months. For instance, experiencing food insecurity for 12 months may expose an individual to prolonged stress due to insufficient resources for food, which may negatively impact mental health and increase reliance on assistance programs for coping, whereas a food insecurity experience for 30 days may not be long enough to initiate a report of poor mental health or to start using food assistance programs. Therefore, we hypothesized that social isolation potentially mediates the cross-sectional relationship of food insecurity with depressive symptoms and resource use in both the short- and long-term frames of reference, suggesting that food insecurity is related to depressive symptoms and using resources when there is social isolation (Figure 1). By investigating these interrelated factors through the conceptual model of the SPM, this research aims to provide insights that could inform interventions targeted at addressing mental health and food insecurity together through programs that improve social connectedness.

## 2. Materials and Methods

### 2.1. Participants and Study Design

This study was a secondary analysis that piloted the evaluation of the relationships described using cross-sectional baseline data from two implementations of the Reaching Rural Veterans (RRV) program. Rural–Urban Continuum codes were used to identify 8 rural counties with rurality scores of 5 or higher in Indiana (May 2021) for the first RRV implementation and 15 rural counties with rurality scores of 4 or higher each in Indiana, Ohio, and Illinois for the second RRV implementation (September 2021) [22]. Rural faith-based pantries from the identified counties were invited and recruited to serve as local partners for the study. Each of the 23 recruited rural faith-based food pantries received funding ($2500) to help implement special RRV outreach events targeting veterans to connect them to resources such as behavioral or medical healthcare, housing assistance, state or government benefits, and others [23,24]. Communities were supported in learning about veteran culture through educational training and ongoing conversations regarding local needs and resources as they devised and tailored plans for implementing RRV outreach events. At each outreach event, representatives from community resources or programs had tables set up to share information with veterans. All veterans who attended were welcomed to participate in the outreach events, but invitations to be included in the study were limited to those who served in the active or reserve component of the U.S. military, were English-speaking, were ≥18 years, visited one of the participating food pantries during the study period, and were willing to participate [24]. Adult (≥18 years) veterans who were interested in being included in the study were given a packet containing a cover letter, two copies of the consent form, and a baseline survey, which included a demographic characteristics questionnaire, measures to assess food security over the past 12 months and over the past 30 days, social isolation, depressive symptoms, and various types of resource use. Veterans were given the option to complete the survey at the RRV events in person or to take it and complete it from home and then mail it back. The participants included in the results featured in this secondary analysis were those who had complete food security information with a reference period of 12 months and 30 days and had complete social isolation information. Purdue University’s Institutional Review Board approved (IRB #: IRB-2021-1467) all study protocols, and participants signed the informed consent forms before beginning study procedures.

### 2.2. Measures

Participants self-completed a demographic questionnaire about personal and household characteristics, including sex, age, marital status, race/ethnicity, education, employment status, household income, number of adults and number of children in the household, stable housing, military status, military branch, years of service, health conditions including obesity, high blood pressure, high cholesterol, and diabetes, approval to use VA healthcare, and service-related disabilities.

Food security was assessed for two reference periods of 30 days and 12 months to capture shorter- and longer-term food security experiences and their relationships with the outcome indicators in this study. For the 30-day reference period, food security was measured using the U.S. Household Food Security Survey Module (U.S. HFSSM), comprising 18 questions that can be modified in terms of the reference period for different study purposes [25,26]. The 18 questions of the U.S. HFSSM are ordered according to the increasing severity of the food security situation, so questions about times of less severely restricted food access are asked first, followed by querying progressively more severely restricted food access situations [26]. In this way, questions about worrying about food proceed to more severe questions asking about changes to diet and eating patterns [26]. Participants usually answer the questions affirmatively (yes) until they reach a more severe food security situation than they have experienced, after which they answer “no” since the following questions ask about more severe situations [26]. For the 12-month reference period, the six-item short form of the U.S. HFSSM was used to reduce respondent burden. Missing responses were imputed following the United States Department of Agriculture (USDA)’s imputation guidance [26]. For the 18-item tool, imputations were carried out only if three or fewer questions were missing for households with children and two or fewer questions for households without children. For the 6-item measure, imputations were completed only when there was one missing response. For both the 6-item and 18-item measures, if the first or the last response was missing, it was imputed as negative (no) to be conservative. Affirmative responses were tallied to determine household food security for both reference periods, and food security status was determined from the total scores and categorized as food secure, or having no changes in food types or amount, for a score of 0–1 for the 6-item measure and 0–2 for the 12-month food security measure. Food insecurity was determined when there were changes either to the food types or amount because of not enough resources for a score ≥ 2 for the 6-item and ≥ 3 for the 18-item measure [26].

Social isolation was assessed using the Patient-Reported Outcomes Measurement Information System (PROMIS) Short Form v2.0—Social Isolation 4a [27]. Developed by the National Institute of Health (NIH), PROMIS is a validated and reliable tool that measures self-reported social isolation using 4 questions with 5 response options: never, rarely, sometimes, usually, and always, which are assigned a value of 1 through 5, respectively [28,29]. To calculate the total social isolation score, individual response options are tallied, and the sum is multiplied by the total number of items in the short form and then divided by the number of items that were answered; the scores are then standardized as a T score with a mean of 50 and a standard deviation of 10 [30]. The total score is calculated for people with complete data only, with the lowest score being 34.0, corresponding to answering “never” for all the questions, and the maximum score being 74.2, corresponding to answering “always” for all the questions [30]. The higher the score, the more socially isolated a person is [30].

Depressive symptoms were assessed using the Patient Health Questionnaire-2 (PHQ-2), a two-question measure asking about how often one is bothered by having little interest or pleasure in doing things and feeling down, depressed, or hopeless over the last 2 weeks [31]. Each question has 4 response options: not at all, several days, more than half the days, and nearly every day [31]. The response options are assigned a value ranging from 0 to 3, and the responses are tallied to calculate the total score per person so that the minimum score is 0 and the maximum is 6 [31]. Participants were considered to have depressive symptoms if they had a score of 3 or more [31].

Resource use was determined by asking participants to answer yes or no to using various types of programs and assistance in the past 12 months [23,24]. Programs included pension and retirement income, income assistance, food assistance resources, and VA benefits. Due to low cell count, some income assistance and food assistance variables were combined into one variable. The pension and retirement income variable included the indication of receiving or not receiving income assistance from any of these programs: VA veterans pension [32], payments from an employer pension [33], retirement fund [34], and social security [35]; this variable had a lot of missing data (missing *n* = 157). The income assistance variable was the indication of receiving or not receiving income from any of the following: TANF [9], unemployment compensation [36], general assistance [37], or assistance from the township trustee [37]. Food assistance programs were categorized into 2 variables; the first variable was constructed as an indication of using SNAP or not, which had a lot of missing data (missing *n* = 134), and the second variable was constructed as an indication of use or no use of any of these programs: the Supplemental Nutrition Assistance Program for Women, Infants, and Children (WIC) [38]), free meals (such as Meals on Wheels America [39], senior centers [40], The Emergency Food Assistance Program (TEFAP) or soup kitchens [41]), and the National School Lunch Program [42]). VA benefits were also combined as one variable, indicating the use or no use of any of these programs: VA healthcare, veteran’s disability benefits, and any other VA benefits such as GI Bill benefits, home loan guarantees, etc. [32]. The variables with missing responses were imputed as negative responses to be conservative. Sensitivity analysis was carried out to determine if imputations would lead to different results (*p* < 0.05 in one case and *p* > 0.05 in another case and vice versa) for the two resource use variables. Sensitivity analysis showed no difference in the significance of *p*-values for all resource use variables between the imputed and non-imputed data in all models except for the use or no use of any of these programs: free meals (e.g., “Meals on Wheels”, senior center, soup kitchen), WIC, free or reduced-price meals at school or childcare within the 12-month food insecurity model, which showed a significant association for non-imputed data (OR = 0.46, *p* = 0.05) but no association for imputed data (Supplementary Table S1). Therefore, imputed data were used for resource use variables in all models, but for the one variable that had different results for imputed data and non-imputed data, the results should be interpreted with caution.

### 2.3. Statistical Analysis

**Figure 2:**
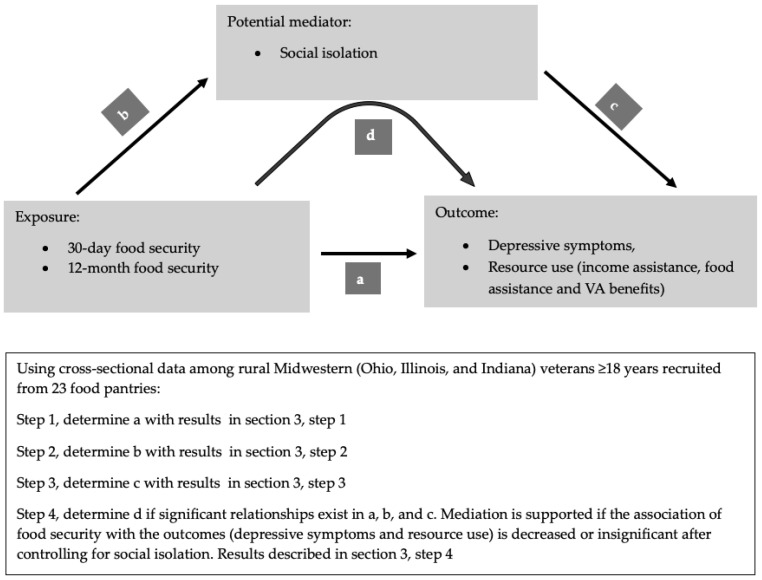
Application of the Baron–Kenny mediation model and hypothesized relationships of the food security association mediated by social isolation to depressive symptoms and resource use using cross-sectional data of rural Midwestern veterans. a = the association of food security with depressive symptoms and resource use, b = the association of food security with social isolation, c = the association of social isolation and depressive symptoms and resource use, and d = the mediation of social isolation in the food security association with depressive symptoms and resource use.

The characteristics of the sample were compared by food security status, separately for 30-day and also for 12-month food security, using chi-square. This analysis identified characteristics that significantly differed among the groups, which could be potential confounders and should be evaluated for inclusion in the final model. Next, the Baron–Kenny mediation approach [43] was used to determine whether social isolation mediated the relationship between food security, depressive symptoms, and resource use (Figure 2).

Multiple logistic regression models were used to evaluate the relationships between 30-day and 12-month household food security with two outcomes, each in separate models: depressive symptoms and the various types of resource use. Multiple linear regression was used to evaluate the relationship between 30-day and 12-month food security with social isolation following the steps described in Figure 2. Logistic regression models were also used to analyze the association of social isolation with depressive symptoms and resource use. Demographic characteristics that significantly varied by food security status were examined for multicollinearity and, if not present, were included in the regression models as covariates. However, some covariates were removed from the covariate list due to various reasons; obesity was removed because it had a large amount of missing data (missing *n* = 142), while stable housing and military branch and years of service were removed because they had low cell count (<10) for some categories. Although there was no indication of multicollinearity between the number of adults in the household and marital status, the number of adults was removed from the list because it did not appear significant in any of the models, and the two variables are conceptually related since being married may determine the number of adults in the household.

## 3. Results

A total of 530 rural veterans participated in the two RRV programs, but 413 were included in this study because they had complete data for social isolation and both 30-day and 12-month food security after imputation. The mean depressive symptoms score and social isolation for the participants in this study are 1.2 and 45.6, respectively (Supplementary Table S2). Those who reported food insecurity for the past 30 days and the past 12 months had a mean depressive symptoms score of 2 and 1.9, respectively, compared to 0.8 and 0.7 for their food-secure counterparts (Supplementary Table S2). The average social isolation score for those who reported 30-day and 12-month food insecurity was 51 and 49.8, respectively, compared to 41.9 and 41.4 for those who were food secure (Supplementary Table S2). The majority of participants were men (85.8%), 18–64 years old (50.1%), married or living with a partner (64.2%), white (90.7%), and had some post-high-school education or above (56.1%) (Table 1). Most were unemployed or out of the labor force (68.2%), reported a household income of $35,000 or less (68.2%), lived in households with two adults (55.3%), had no children (78.1%), and reported having stable housing over the past 12 months (93.9%) (Table 1). A larger proportion were veterans (91.6%) who served in the Army (53.7%), did not serve in the Guard or Reserve (58.5%), and had served for 10 years or less (81.1%) (Table 1). Additionally, most participants reported no obesity (69.0%), had one or more of the following health conditions: high blood pressure, high cholesterol, or diabetes (70.2%), were approved to receive Veterans Affairs healthcare (71.4%), and did not have any service-related disability recognized (61.5%) or unrecognized (73.9%) by the VA (Table 1).

The chi-squared results show that food security status for both 30-day and 12-month indicators was statistically related to the distribution of certain demographic characteristics. Regardless of the 30-day or 12-month food security measure, compared to food-secure participants, food-insecure participants were mostly 18–64 years old (69.6%, 63.4%), married (57.8%, 57.2%), with a household income of $20,0000 or less (50.3%, 47.4%), from households with two adults (43.1%, 46.3%) and no children (65.4%, 70.3), reported having stable housing (88%, 90.1%), served in the army (61.5%, 59.7%), reported no obesity (59.1%, 60%), and reported no service-related disability unrecognized by the VA (67.7%, 67.5%), with depressive symptoms in 26.8% and 25.1% of participants for 30-day and 12-month food security, respectively (all *p* < 0.05) (Table 1 and Table 2). In addition, those who reported food insecurity in the past 30 days were mostly unemployed or not in the labor force (79.6%) (Table 1), while those who reported food insecurity in the past 12 months were mostly male (81.7%) and reported having served for 10 years or less (85.9%) (*p* < 0.05) (Table 2).

The logistic and multiple linear regression were performed to address the research objectives. The results of Step 1 showed that veterans who reported being food insecure in the past 30 days had higher odds of depressive symptoms (odds ratio (OR) = 2.28, *p* = 0.02), receiving income from 1 or more of these programs: VA veterans pension, payments from an employer pension, withdrawals from a retirement fund, and Social Security Income (OR = 2.27, *p* = 0.03), and receiving income from any of these programs: TANF, unemployment compensation, general assistance, or assistance from the township trustee (OR = 3.92, *p* = 0.01) (Table 3). Those who reported being food insecure in the past 12 months were more likely to report depressive symptoms (OR = 2.34, *p* = 0.03), to be receiving income from any of these programs: VA veterans pension, payments from an employer pension, withdrawals from a retirement fund, or Social Security Income (OR = 2.51, *p* = 0.01), to be receiving income from any of these programs: TANF, unemployment compensation, general assistance, or assistance from the township Trustee (OR = 3.09, *p* = 0.04), and less likely to be enrolled in any VA benefits program (OR = 0.55, *p* = 0.03) (Table 3). The results further showed that food-insecure veterans were less likely to be enrolled in any of these programs: free meals (e.g., “Meals on Wheels”, senior center, soup kitchen), Women, Infants, and Children program (WIC), free or reduced-price meals at school or childcare (OR = 0.46, *p* = 0.05) (Supplementary Table S1) when the data for this outcome was not imputed but there was no significant association after imputation for 12-month food security (Table 3).

Next, the results of Step 2 showed that veterans who reported being food insecure both in the past 30 days and 12 months had 7.2 and 6 points higher social isolation scores, respectively (*p* < 0.0001) (Table 4).

Following this, the analysis of Step 3 indicated that socially isolated veterans were more likely to report depressive symptoms after controlling for 30-day or 12-month food security status, respectively (OR = 1.13, *p* < 0.0001; OR = 1.12, *p* < 0.0001) (Table 5). No associations were observed between social isolation and resource use after controlling for 30-day or 12-month food security.

Finally, for Step 4, since there were significant relationships in steps 1 and 2 between food security and depressive symptoms, food security and social isolation, and between social isolation and depressive symptoms, logistic regression was completed, modeling the relationship between 30-day and 12-month food security and depressive symptoms while controlling for social isolation in the model. The association of food security with depressive symptoms was not significant for both 30-day and 12-month food security (Table 6), supporting that social isolation mediates this association.

## 4. Discussion

This study evaluated the SPM-based conceptualization of social isolation as a potential mediator in the relationship of 30-day and 12-month food insecurity with depressive symptoms and resource use among rural veterans using cross-sectional data. Applying the Baron–Kenny mediation analysis provided evidence supporting the hypothesis that social isolation mediates the association of food security with depressive symptoms when these factors were measured at a certain time point in this unique group, but there was no evidence of mediation in the relationship of food security with resource use.

Social isolation may be implicated in the negative relationship of both short-term and long-term food insecurity on mental health in rural veterans. Evidence of social isolation mediation in the food security relationship with depressive symptoms for both 30-day and 12-month food security using a cross-sectional framework highlights the importance of social networks in mental health well-being regardless of whether food security is shorter or longer term. Previous literature has demonstrated an association between food insecurity and social isolation [13] and an association between social isolation and negative mental health outcomes, particularly depressive symptoms [44], but the role social isolation may play in the relationship between food insecurity and mental health is a research gap where this study contributes. As the SPM-derived framework demonstrates, food insecurity is conceptualized as a stressor in finding affordable ways to obtain enough food for a household with limited financial resources. Without social support to buffer food insecurity stress and to potentially help, psychological well-being may be at stake. Social isolation may be part of this relationship, particularly for veterans, since military culture emphasizes group cohesion [45], and veterans in rural areas may find it difficult to stay in contact with their military colleagues, making transitioning into civilian society difficult due to limited social support [46]. Social support, either through family and friends or community and government assistance, may contribute to achieving food security and improving mental health [47,48,49,50]. Family and friends, through directly sharing food or offering rides to grocery stores or food pantries and sharing information about community and government resources, could help improve food availability in one’s household [47,48]. Receiving social support may improve mental health by helping to counteract the stress that comes with food insecurity [50]. Social isolation is, therefore, an important factor in how food insecurity may be present in the lives of rural veterans. Creating programs that facilitate social interactions through peer support networks or veteran-specific activities, such as the Reaching Rural Veterans program, an intervention in rural food pantries that brings together service organizations to celebrate and reach out to rural veterans, may help reduce social isolation while simultaneously addressing food insecurity and mental health [23].

Results also revealed significant relationships between food insecurity and the use of various income assistance programs. Veterans who experienced food insecurity over both 30 days and 12 months had higher odds of using one or more pension or retirement resources, including VA veterans pensions, payments from an employer pension, withdrawals from a retirement fund, and Social Security Income, as well as being enrolled in any of these income assistance programs: TANF, Unemployment compensation, General Assistance, or Assistance from the Township Trustee. Experiencing food insecurity for both 30 days and 12 months may prompt help seeking and enrollment in relief programs such as TANF. The significant link between food security and the use of assistance resources suggests that these programs are reaching rural veterans despite their locations. However, even though these resources are utilized, persistent food insecurity suggests that current support may not be enough for rural veterans. On the other hand, veterans who reported experiencing food insecurity in the past 12 months were 42% less likely to use VA benefits compared to their food-secure counterparts. Research shows that veterans have limited awareness about VA benefits in general [32] and, together with the unique challenges that come with residing in rural areas [51], may contribute to the decreased use of VA benefits among food-insecure rural veterans. The mixed results and lack of mediation of social isolation in the relationship between food insecurity and resource use suggest that factors other than limited financial resources may determine the use of assistance programs, which warrants further research to determine those factors in this population. These findings suggest that there are potential gaps in awareness and how effectively current assistance programs address the specific needs of veterans living in rural settings.

### 4.1. Strengths and Limitations

A strength of this study is the use of a unique sample of rural veterans, who are not often included in studies. The use of two reference periods for food security, including 12 months and 30 days, provides insights into food insecurity experiences over a shorter and longer amount of time among rural veterans, which is also another strength of this study. However, there are limitations as well. Causal inference may not be made based on the results because cross-sectional data were used to investigate the mediation of social isolation in the relationship of food security and depressive symptoms. Therefore, further studies should be carried out to evaluate causal mediation and other methods, including bootstrap, the Sobel test, and Structural Equation Modeling may enhance statistical robustness [52]. There was also no investigation of whether those who reported 30-day food insecurity were already experiencing food insecurity previously; therefore, results should be interpreted accordingly. The measures for social isolation and depressive symptoms were both short-form screening tools and not comprehensive. Similarly, the 6-item food security measure for the 12-month reference period may be less precise or reliable compared with the 18-item measure but was selected to reduce user burden. Collecting data using a mail-in approach may have contributed to low response rates, hence a high amount of missing data. Imputation was performed to retain statistical power, coding the missing responses as negative to be conservative. A sensitivity analysis was performed, and the results were mostly consistent for both imputed and unimputed datasets, rendering the use of imputed data appropriate, yet the reader is reminded that any imputation may introduce potential bias and variability. Recruitment of a convenience sample from food pantries also limits the generalizability of the results to all rural veterans and other groups, including disabled people, elderly people, and the general population, since the veterans visiting food pantries and interested in participating in a study may differ from those who are not and may not fully represent these groups. Future studies should consider in-person data collection methods to ensure high response rates and recruit rural veterans from diverse recruitment sites to enhance generalizability. The rural veteran population in these Midwestern states may not be similar to those groups in other regions of the U.S., and there may be other factors influencing the relationship between food security and resource use that should be further evaluated in the future; for example, trauma history, access to healthcare, and community infrastructure. Longitudinal evaluation of the role of social isolation and other factors, including access to transportation, the internet, etc., in the relationship of food insecurity with mental health and resource use is recommended for future studies to more rigorously evaluate the relationships explored here.

### 4.2. Implications for Policy and Practice

The study’s findings have several implications for policy and practice. Designing interventions that encourage community involvement through veteran-specific events and support networks may help alleviate food insecurity and its relationship to depressive symptoms among rural veterans, offering a more comprehensive approach to veteran well-being [23,53]. Peer support groups and community-based programs could provide a threefold benefit by addressing social isolation, food insecurity, and mental health among rural veterans [54]. Lastly, tailoring assistance programs to serve the needs of rural veterans better could improve their utilization and effectiveness [22]. This might include expanding mobile services, creating more accessible program locations, reducing stigma, and increasing program awareness through community education and veteran-centered outreach.

## 5. Conclusions

This study highlights the need for a holistic approach to addressing food insecurity among rural veterans. An approach that addresses improving food security and social isolation may be particularly helpful to mental health among rural veterans. Acknowledging and targeting social isolation, policies, and interventions that integrate peer support and community outreach to promote food security could support rural veterans food security and mental health.

## Figures and Tables

**Table 1:** Demographic characteristics by 30-day food security among rural Midwestern (Ohio, Illinois, and Indiana) veterans ≥ 18 years recruited from 23 food pantries in May and September 2021 (n = 413).

Characteristics		N (%)			*p*-Value ^1^
		Total	Food Secure	Food Insecure	
Sex					
	Male	352 (85.5)	220 (88.3)	132 (82.0)	0.07
	Female	58 (14.2)	29 (11.6)	29 (18.0)	
Age					
	18–64 years	206 (50.1)	94 (37.6)	112 (69.6)	<0.0001 *
	≥65 years	205 (49.9)	156 (62.4)	49 (30.4)	
Marital status					
	Married/living with partner	263 (64.2)	170 (68.3)	93 (57.8)	0.03 *
	Widowed/divorced/separated/never married	147 (35.8)	79 (31.7)	68 (42.2)	
Race/ethnicity					
	White	369 (90.7)	225 (91.5)	144 (89.4)	0.58
	African American	14 (3.4)	9 (3.7)	5 (3.1)	
	Hispanic	6 (1.5)	4 (1.6)	2 (1.2)	
	Asian	1 (0.2)	1 (0.4)	0 (0.0)	
	Indian American	12 (2.9)	5 (2.0)	7 (4.3)	
	Native American	5 (1.2)	2 (0.8)	3 (1.9)	
Race (grouped into 2 categories)					
	White	369 (90.7)	225 (91.5)	144 (89.4)	0.49
	Other	38 (9.3)	21 (8.5)	17 (10.6)	
Education					
	High school, equivalent or less	173 (43.5)	98 (40.5)	75 (48.1)	0.14
	Some post-high-school education and above	225 (56.1)	144 (59.5)	81 (51.9)	
Employment status					
	Employed	77 (20.3)	47 (20.2)	30 (20.4)	0.01 *
	Not employed	44 (11.6)	18 (7.7)	26 (17.7)	
	Not in labor force	259 (68.1)	168 (72.1)	91 (61.9)	
Household income in the last 12 months					
	≤$20,000	127 (33.1)	52 (23.3)	75 (50.3)	<0.0001 *
	$21,000–$35,000	129 (34.7)	80 (35.9)	49 (32.9)	
	>$35,000	116 (31.2)	91 (40.8)	25 (16.8)	
Number of adults					
	1	94 (23.0)	58 (23.3)	36 (22.5)	<0.0001 *
	2	226 (55.2)	157 (63.1)	69 (43.1)	
	≥3	89 (21.8)	34 (13.6)	55 (34.4)	
Number of children					
	0	318 (78.1)	214 (86.3)	104 (65.4)	<0.0001 *
	≥1	89 (21.9)	34 (13.7)	55 (34.6)	
Stable housing over the past 12 months					
	Yes	384 (93.9)	243 (97.6)	141 (88.1)	0.0001 *
	No	26 (6.1)	6 (2.4)	19 (11.9)	
Military status					
	Veteran	351 (91.6)	215 (91.9)	136 (91.3)	0.83
	Active/non-active	32 (8.2)	19 (8.1)	13 (8.7)	
Military Branch					
	Air Force	63 (15.4)	50 (20.1)	13 (8.1)	0.01 *
	Army	220 (53.7)	121 (48.6)	99 (61.5)	
	Coast Guard	3 (0.7)	3 (1.2)	0 (0.0)	
	Marine Corps	41 (10.0)	24 (9.6)	17 (10.5)	
	Navy	75 (18.3)	46 (18.5)	29 (18.0)	
	Multiple branches	8 (1.9)	5 (2.0)	3 (1.9)	
Guard/Reserve					
	Yes	154 (41.5)	97 (40.9)	57 (42.5)	0.76
	No	217 (58.5)	140 (59.1)	77 (57.5)	
Years of service					
	1–10 years	318 (81.1)	189 (79.1)	129 (84.3)	0.34
	11–20 years	42 (10.7)	27 (11.3)	15 (9.8)	
	21–40 years	32 (8.2)	23 (9.6)	9 (5.9)	
Obesity					
	Yes	84 (31.0)	46 (25.8)	38 (40.9)	0.03 *
	No	187 (69.0)	132 (74.2)	55 (59.1)	
Any of the following conditions: High blood pressure High cholesterol Diabetes					
	Yes	233 (70.2)	144 (68.9)	89 (72.4)	0.50
	No	99 (29.8)	65 (31.1)	34 (27.6)	
Approved to receive Veterans Affairs healthcare					
	Yes	275 (71.4)	175 (74.8)	100 (66.2)	0.07
	No	110 (28.6)	59 (25.2)	51 (33.8)	
Service-related Veterans Affairs-recognized disability					
	Yes	158 (38.4)	94 (37.9)	64 (39.5)	0.74
	No	252 (61.5)	154 (62.1)	98 (60.5)	
Service-related disability not recognized by Veterans Affairs					
	Yes	103 (26.1)	53 (22.1)	50 (32.3)	0.03 *
	No	292 (73.9)	187 (77.9)	105 (67.7)	
Depressive symptoms					
	Yes	68 (17.5)	27 (11.5)	41 (26.8)	0.0001 *
	No	320 (82.5)	208 (88.5)	112 (73.2)	

^1^ The total numbers may not add up to the total n, and the percentages may not add up to 100 due to missing data and rounding. * Denotes statistically significant results at ≤0.05.

**Table 2:** Demographic characteristics by 12-month food security among rural Midwestern (Ohio, Illinois, and Indiana) veterans ≥ 18 years recruited from 23 food pantries in May and September 2021 (n = 413).

Characteristics		N (%)			*p*-Value ^1^
		Total	Food Secure	Food Insecure	
Sex					
	Male	352 (85.8)	187 (89.9)	165 (81.7)	0.02 *
	Female	58 (14.2)	21 (10.1)	37 (18.3)	
Age					
	18–64	206 (50.1)	78 (37.3)	128 (63.4)	<0.0001 *
	≥65	205 (49.9)	131 (62.7)	74 (36.6)	
Marital status					
	Married/living with partner	263 (64.1)	148 (70.8)	115 (57.2)	0.004 *
	Widowed/Divorced/Separated/Never married	147 (35.8)	61 (29.2)	86 (42.8)	
Race/Ethnicity					
	White	369 (90.7)	186 (90.7)	183 (90.6)	0.56
	African American	14 (3.4)	8 (3.9)	6 (3.0)	
	Hispanic	6 (1.5)	4 (1.9)	2 (1.0)	
	Asian	1 (0.2)	1 (0.5)	0 (0.0)	
	Indian American	12 (2.9)	4 (1.9)	8 (4.0)	
	Native American	5 (1.2)	2 (1.0)	3 (1.5)	
Race					
	White	369 (90.7)	186 (90.7)	183 (90.6)	0.96
	Other	38 (9.3)	19 (9.3)	19 (9.4)	
Education					
	High school, equivalent or less	173 (43.5)	82 (40.6)	91 (46.4)	0.38
	Some post-high-school education and above	225 (56.5)	120 (59.4)	105 (53.6)	
Employment status					
	Employed	77 (20.3)	43 (21.7)	34 (18.7)	0.08
	Not employed	44 (11.6)	16 (8.1)	28 (15.4)	
	Not in labor force	259 (68.1)	139 (70.2)	120 (65.9)	
Household income in the last 12 months					
	≤$20,000	127 (34.1)	37 (20.3)	92 (47.4)	<0.0001 *
	$21,000–$35,000	129 (34.7)	57 (31.3)	75 (37.9)	
	>$35,000	116 (31.2)	88 (48.3)	31 (14.7)	
Number of adults					
	1	94 (23.0)	45 (21.6)	49 (24.4)	0.0002 *
	2	226 (55.2)	133 (63.9)	93 (46.3)	
	≥3	89 (21.8)	30 (14.4)	59 (29.3)	
Number of children					
	0	318 (78.1)	178 (85.6)	140 (70.3)	0.0002 *
	≥1	89 (21.9)	30 (14.4)	59 (29.6)	
Stable housing over the past 12 months					
	Yes	384 (93.9)	203 (97.6)	181 (90.1)	0.001 *
	No	25 (6.1)	5 (2.4)	20 (9.9)	
Military status					
	Veteran	351 (91.6)	178 (91.7)	173 (91.5)	0.94
	Active/Non-active	32 (8.4)	16 (8.2)	16 (8.5)	
Military Branch					
	Air Force	63 (15.4)	46 (22.0)	17 (8.5)	0.02 *
	Army	220 (53.7)	100 (47.8)	120 (59.7)	
	Coast Guard	3 (0.7)	2 (1.0)	1 (0.5)	
	Marine Corps	41 (10.0)	20 (9.6)	21 (10.4)	
	Navy	75 (18.3)	38 (18.2)	37 (18.4)	
	Multiple branches	8 (1.9)	3 (1.4)	5 (2.5)	
Guard/Reserve					
	Yes	154 (41.5)	88 (44.0)	66 (38.6)	0.29
	No	217 (58.5)	112 (56.0)	105 (61.4)	
Years of service					
	1–10	318 (81.1)	153 (76.5)	165 (85.9)	0.05 *
	11–20	42 (10.7)	26 (13.0)	16 (8.3)	
	21–40	32 (8.2)	21 (10.5)	11 (5.7)	
Obesity					
	Yes	84 (31.0)	34 (23.3)	50 (40)	0.003 *
	No	187 (69.0)	112 (76.7)	75 (60.0)	
Any of the following conditions: High blood pressure High cholesterol Diabetes					
	Yes	233 (70.2)	123 (69.5)	110 (71.0)	0.77
	No	99 (29.8)	54 (30.5)	45 (29.0)	
Approved to receive Veterans Affairs healthcare					
	Yes	275 (71.4)	141 (72.7)	134 (70.2)	0.58
	No	110 (28.5)	53 (27.3)	57 (29.8)	
Service-related Veterans Affairs-recognized disability					
	Yes	158 (38.4)	80 (38.6)	78 (38.4)	0.96
	No	22 (61.5)	127 (61.3)	125 (61.6)	
Service-related disability not recognized by Veterans Affairs					
	Yes	103 (26.1)	40 (19.9)	63 (32.5)	0.004 *
	No	292 (73.9)	161 (80.1)	131 (67.5)	
Depressive symptoms					
	Yes	68 (17.5)	20 (10.1)	48 (25.1)	<0.0001 *
	No	320 (82.5)	177 (89.8)	143 (74.9)	

^1^ The total numbers may not add up to the total n, and the percentages may not add up to 100 due to missing data and rounding. * Denotes statistically significant results at ≤0.05.

**Table 3:** Odds ratios for logistic regression analysis of the 30-day and 12-month food security status (food-insecure compared to food-secure) on depressive symptoms and resource use among rural Midwestern (Ohio, Illinois, and Indiana) veteran adults (n = 413) to address Step 1 of the Barron Kenny mediation analysis.

Outcome		Odds Ratio	Standard Error	*p*-Value ^1^
30-day food security model				
Depressive symptoms	Yes	2.28	0.3	0.02 *
Enrollment in 1 or more VA benefits	Yes	0.60	0.3	0.07
Receiving income from any of these programs: VA veterans’ pension, Payments from an employer pension, Withdrawals from a retirement fund, Social Security Income	Yes	2.27	0.4	0.03 *
Receiving income from any of these programs: TANF, Unemployment compensation, General Assistance, or Assistance from the Township Trustee	Yes	3.92	0.5	0.01 *
Enrollment in SNAP	Yes	1.58	0.3	0.15
Enrollment in any of these programs: free meals (e.g., “Meals on Wheels”, senior center, soup kitchen), Women, Infants, and Children program (WIC), free or reduced-price meals at school or childcare	Yes	0.78	0.4	0.50
12-month food security model				
Depressive symptoms	Yes	2.34	0.4	0.02 *
Enrollment in 1 or more VA benefits	Yes	0.55	0.3	0.03 *
Receiving income from any of these programs: VA veterans’ pension, Payments from an employer pension, Withdrawals from a retirement fund, Social Security Income	Yes	2.51	0.3	0.01 *
Receiving income from any of these programs: TANF, Unemployment compensation, General Assistance, or Assistance from the Township Trustee	Yes	3.09	0.5	0.04 *
Enrollment in SNAP	Yes	1.30	0.3	0.41
Enrollment in any of these programs: free meals (e.g., “Meals on Wheels”, senior center, soup kitchen), Women, Infants, and Children program (WIC), free or reduced-price meals at school or childcare	Yes	0.70	0.4	0.34

^1,^* Denotes statistically significant results at ≤0.05.

**Table 4:** Results of the multiple linear regression analysis of 30-day and 12-month food security status (food-insecure compared to food-secure) on social isolation among rural Midwestern (Ohio, Illinois, and Indiana) veteran adults (n = 413) to address Step 2 of the Barron Kenny mediation analysis.

Outcome	β Coefficient	Standard Error	*p*-Value ^1^
30-day food security			
Social isolation	7.2	1.2	<0.0001 *
12-month food security			
Social isolation	6.0	1.1	<0.0001 *

^1,^* Denotes statistically significant results at ≤0.05.

**Table 5:** Odds ratios for logistic regression analysis of social isolation on depressive symptoms and resource use accounting for 30-day and 12-month food security among rural Midwestern (Ohio, Illinois, and Indiana) veteran adults (n = 413) to address Step 3 of the Barron Kenny mediation analysis.

Outcome		Odds Ratio	Standard Error	*p*-Value ^1^
Model controlling for 30-day food security				
Depressive symptoms	Yes	1.13	0.02	<0.0001 *
Receiving income from any of these programs: VA veterans pension, Payments from an employer pension, Withdrawals from a retirement fund, Social Security Income	Yes	1.02	0.02	0.40
Receiving income from any of these programs: TANF, Unemployment compensation, General Assistance, or Assistance from the Township Trustee	Yes	1.02	0.02	0.33
Model controlling for 12-month food security				
Depressive symptoms	Yes	1.12	0.02	<0.0001 *
Enrollment in 1 or more VA benefits	Yes	1.01	0.01	0.62
Receiving income from any of these programs: VA veterans pension, Payments from an employer pension, Withdrawals from a retirement fund, Social Security Income	Yes	1.02	0.02	0.22
Receiving income from any of these programs: TANF, Unemployment compensation, General Assistance, or Assistance from the Township Trustee	Yes	1.03	0.02	0.22

^1,^* Denotes statistically significant results at ≤0.05.

**Table 6:** Odds ratios for logistic regression analysis of 30-day and 12-month food security (food-insecure compared to food-secure) on depressive symptoms accounting for social isolation among rural Midwestern (Ohio, Illinois, and Indiana) veteran adults (n = 413) to address Step 4 of the Barron Kenny mediation analysis.

Outcome		Odds Ratio	Standard Error	*p*-Value
30-day food security				
Depressive symptoms	Yes	0.92	0.40	0.85
12-month food security				
Depression	Yes	1.06	0.41	0.90

## Data Availability

Data are unavailable due to privacy or ethical restrictions. Requests to access the datasets should be directed to heicherm@purdue.edu.

## Supplementary Materials

The following supporting information can be downloaded at: https://www.mdpi.com/article/10.3390/nu17020318/s1, Table S1: Sensitivity analysis showing imputed data and non-imputed data odds ratios for logistic regression analysis of the 30-day food security status, 12-month food security status (food insecure compared to food secure), resource use variables whose missing responses were imputed among rural midwestern (Ohio, Illinois, and Indiana) veteran adults (*n* = 413) to address Steps 1 and 2 of the Barron Kenny mediation analysis; Table S2: Depressive symptoms and social isolation percentages and mean scores among rural midwestern (Ohio, Illinois, and Indiana) veteran adults (*n* = 413) to address Step 4 of the Barron Kenny mediation analysis.
