# Supplementary material for: A Cross-Sectional Study Exploring the Role of Social Isolation in the Relationship Between Food Insecurity, Depressive Symptoms, and Resource Use Among Midwestern Rural Veterans in the U.S
Source: Nutrients. 2025 Jan 16;17(2):318. doi: 10.3390/nu17020318 (PMC11769182; doi:10.3390/nu17020318)
Supplement: Supplementary file 1 [file nutrients-17-00318-s001.zip › nutrients-3408178-supplementary.pdf]

Supplementary Materials

Supplementary Table S1. Sensitivity analysis showing imputed data and non-imputed data odds ratios for logistic regression analysis of the 30-day food security status, 12-month food security status (food insecure compared to food secure), resource use variables whose missing responses were imputed among rural midwestern (Ohio, Illinois, and Indiana) veteran adults (*n* = 413) to address Steps 1 and 2 of the Barron Kenny mediation analysis.

**Outcome**		**Odds ratio**	**Standard Error**	**P-value**[1]
	30-day food security model			
Receiving income from any of these programs: TANF, Unemployment compensation, General Assistance, or Assistance from the Township Trustee (Imputed data)	Yes	3.92	0.5	0.01*
Receiving income from any of these programs: TANF, Unemployment compensation, General Assistance, or Assistance from the Township Trustee (Non-imputed data)	Yes	5.00	0.5	0.003*
Enrollment in SNAP (Imputed data)	Yes	1.58	0.3	0.15
Enrollment in SNAP (Non-imputed data)	Yes	1.52	0.4	0.25
Enrolment in any of these programs: Free meals (e.g. "Meals on Wheels", senior center, soup kitchen), Women, Infants, and Children program (WIC), Free or reduced price meals at school or childcare (Imputed data)	Yes	0.78	0.4	0.50
Enrolment in any of these programs: Free meals (e.g. "Meals on Wheels", senior center, soup kitchen), Women, Infants, and Children program (WIC), Free or reduced price meals at school or childcare (Non-imputed data)	Yes	0.63	0.4	0.26
	12-month food security model			
Receiving income from any of these programs: TANF, Unemployment compensation, General Assistance, or Assistance from the Township Trustee (Imputed data)	Yes	3.09	0.5	0.04*

Receiving income from any of these programs: TANF, Unemployment compensation, General Assistance, or Assistance from the Township Trustee (Non-imputed data)	Yes	4.03	0.6	0.02*
Enrollment in SNAP (Imputed data)	Yes	1.30	0.3	0.41
Enrollment in SNAP (Non-imputed data)	Yes	1.63	0.4	0.17
Enrolment in any of these programs: Free meals (e.g. "Meals on Wheels", senior center, soup kitchen), Women, Infants, and Children program (WIC), Free or reduced price meals at school or childcare (Imputed data)	Yes	0.70	0.4	0.34
Enrolment in any of these programs: Free meals (e.g. "Meals on Wheels", senior center, soup kitchen), Women, Infants, and Children program (WIC), Free or reduced price meals at school or childcare (Non-imputed data)	Yes	0.46	0.4	0.05*

[1] * Denotes statistically significant results at ≤0.05.

Supplementary Table S2. Depressive symptoms and social isolation percentages and mean scores among rural midwestern (Ohio, Illinois, and Indiana) veteran adults (n = 413) to address Step 4 of the Barron Kenny mediation analysis.

**Outcome**	**N (%)**	**Total sample**		**30-day food secure**		**30-day food insecure**		**12-month food secure**		**12-month food insecure**	
		Mean	SD[1]	Mean	SD[1]	Mean	SD[1]	Mean	SD[1]	Mean	SD[1]
Depressive symptoms											
Yes	214 (51.8)	1.2	1.6	0.8	1.3	2.0	1.7	0.7	1.2	1.9	1.7
No	61 (14.8)										
Social isolation		45.6	10.8	42.0	9.5	51.0	10.4	41.4	9.2	49.8	10.8

[1] SD: Standard Deviation
